# Acute Attack of Pseudogout with the Wide Lesion in Lumbar Spondylolytic Spondylolisthesis

**DOI:** 10.1155/2020/4512695

**Published:** 2020-07-29

**Authors:** Hironari Kaneyama, Yuichiro Morishita, Osamu Kawano, Takuaki Yamamoto, Takeshi Maeda

**Affiliations:** ^1^Department of Orthopedic Surgery, Spinal Injuries Center, Iizuka, Japan; ^2^Department of Orthopedic Surgery, Fukuoka University, Fukuoka, Japan

## Abstract

**Objective:**

To report a rare case of an acute attack of calcium pyrophosphate dihydrate (CPPD) deposition disease in a patient with lumbar spondylolytic spondylolisthesis, which demonstrated widespread lesion with neurological deficit.

**Methods:**

An 86-year-old woman presented with high fever and bilateral neurological deficit of the lower extremities.

**Results:**

CRP was elevated (20.9 mg/dl). Plain radiographs and computed tomography images showed bilateral L4 spondylolytic spondylolisthesis. Sagittal magnetic resonance (MR) images revealed effusion at the L3-4 interspinous space, and a gadolinium- (GD-) enhanced epidural mass was observed at the level of L4 vertebral body. Axial MR images showed an intra- or epidural lesion at L2-3. Moreover, epidural GD-enhanced masses compressed the dural sac in the shape of a cross at the L3-4 and L4-5 segments. The patient was suspected of having pyogenic arthritis of the lumbar spine in initial diagnosis. A total of 1.2 ml of fluid with a murky, pus-like synovial effusion was aspirated from the L3-4 interspinous space under the fluoroscopic image. Smear speculum of synovial fluid tested negative for bacteria and fungi; however, a number of crystals were seen. Based on the result of smear speculum, we suspected the pathology as crystal deposition disease. Based on polarized light microscopy, which revealed monocle or triclinic intracellular crystals with a positive birefringence, the patient was diagnosed with pseudogout of the lumbar spine. Nonsteroidal anti-inflammatory drugs (NSAIDs) were administered by intravenous drip injection for 3 days, and local and systemic inflammatory signs, as well as neurological deficits, dramatically improved.

**Conclusions:**

We encountered the rare case of an acute attack of pseudogout with the wide lesion in the lumbar spondylolytic spondylolisthesis. Multiple culture of the effusion provided a definitive diagnosis, which allowed for appropriate, minimally invasive treatment for 8 weeks of NSAID administration that provided the satisfactory recovery from the symptoms.

## 1. Introduction

Calcium pyrophosphate dihydrate (CPPD) deposition disease is known as “pseudogout” because of the clinical similarity to gouty arthritis [1]. According to the European League Against Rheumatism, four different clinical presentations can be observed: (1) asymptomatic CPPD, (2) osteoarthritis with CPPD, (3) acute CPP crystal arthritis, and (4) chronic CPP inflammatory crystal arthritis.

CPPD deposition disease commonly involves the major peripheral joints. Although CPPD deposition disease of the spine is less common, it can involve the ligamentum flavum; the longitudinal, supraspinous, and interspinous ligaments; the intervertebral discs; and the sacroiliac and apophyseal joint [2–14]. The definitive diagnosis of CPPD deposition disease is suspected on the basis of the clinical picture and radiographic/laboratory findings. The reference standard for the diagnosis of CPPD is based on the identification of CPP crystals in synovial fluid by light microscopy, compensated polarized light microscopy, or phase contrast microscopy [15].

To the best of our knowledge, few reports have thus far referred to an acute attack of pseudogout in the lumbar spine. In our case, there was a large extent of CPPD crystal deposition in the L2-3-4 ligamentum flavum, the L4 spondylolysis, the L3-4 facet joints, and the L3-4 interspinous space. There are no known reports of such widespread related to CPPD crystal deposition in the lumbar spine. We hereby report an acute attack of pseudogout in L4 spondylolytic spondylolisthesis, which demonstrated widespread lesion with neurological deficits.

## 2. Case Report

An 86-year-old woman presented with sudden low back pain and bilateral lower extremity muscle weakness with numbness. The patient could neither stand alone nor walk unaided. She had bronchitis, with a heavy cough and high fever, a week before the onset of neurological symptoms. The patient had no history of peripheral pseudogout arthritis.

On admission, her body temperature was 37.6°C and she had localized heat and tenderness on her lower back. C-reactive protein (CRP) value was elevated (20.9 mg/dl). Neurological examination was normal for objective sensory evaluation; however, motor function below the iliopsoas was 3 to 4 in manual muscle testing. There were no abnormalities in her deep tendon reflex or pathological reflex.

**Figure 1:**
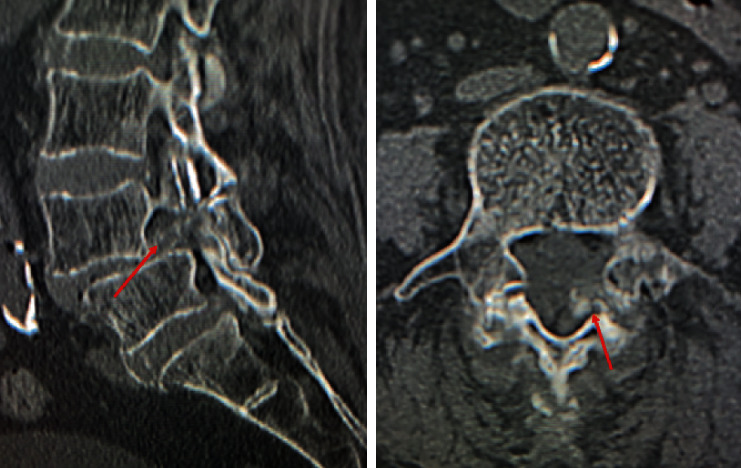
A nodular calcification mass was observed from the location of left L4 spondylolysis to the intraspinal canal in plain CT images.

**Figure 2:**
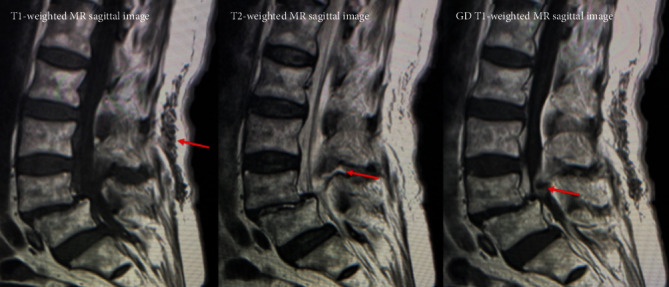
An intensity change of the L1-4 supraspinal ligament in T1-weighted images, effusion at the L3-4 interspinous space in T2-weighted images, and a GD-enhanced epidural mass at the level of L4 vertebral body.

**Figure 3:**
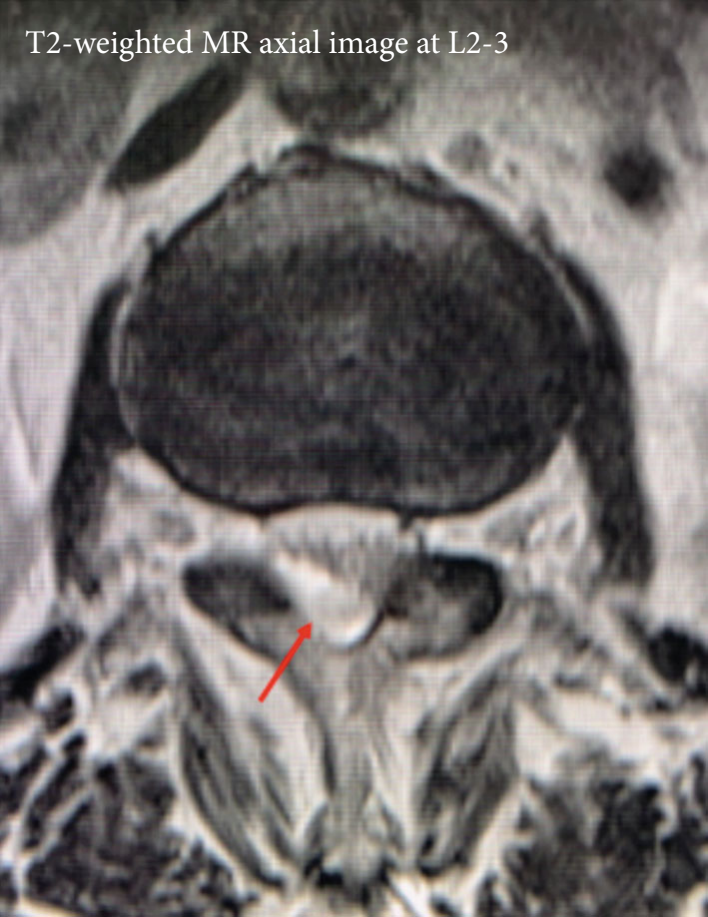
An intra- or epidural lesion at L2-3.

**Figure 4:**
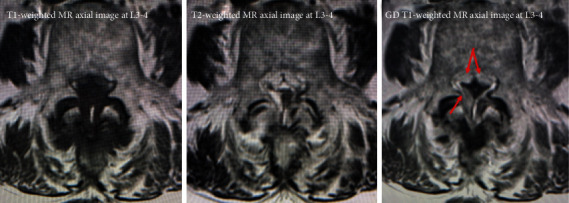
Epidural GD-enhanced masses compressed the dural sac in the shape of across at the L3-4.

Plain radiographs of the lumbar spine showed L4 spondylolisthesis without dynamic instability. Plain computed tomography (CT) images showed bilateral L4 spondylolytic spondylolisthesis. The nodular calcifications were observed at the L3-4 interspinous space, the L2-3 and L3-4 ligamentum flavum, the bilateral L3-4 facet joints, and the location of left L4 spondylolysis (Figure 1). Sagittal magnetic resonance (MR) images (Figure 2) showed an intensity change of the L1-4 supraspinal ligament in T1-weighted images and effusion at the L3-4 interspinous space in T2-weighted images. A gadolinium- (GD-) enhanced epidural mass was observed at the level of L4 vertebral body, which compressed the dural sac. Axial T2-weighted MR images showed an intra- or epidural lesion at L2-3 (Figure 3). Moreover, epidural GD-enhanced masses compressed the dural sac in the shape of a cross at the L3-4 (Figure 4) and L4-5 segments.

**Figure 5:**
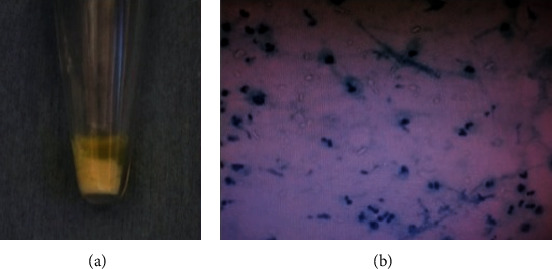
(a) Aspiration of the L3-4 interspinous space yielded a murky, pus-like synovial effusion. (b) Multiple cultures of the synovial fluid tested negative for bacteria and fungi, whereas compensated polarized light microscopy revealed monocle or triclinic intracellular crystal with a positive birefringence.

The patient was suspected of having pyogenic arthritis of the lumbar spine with intra- or epidural abscess in initial diagnosis. Therefore, aspiration of the effusion was performed for the bacterium identification. A total of 1.2 ml of fluid with a murky, pus-like synovial effusion (Figure 5(a)) was aspirated from the L3-4 interspinous space under the fluoroscopic image. Smear speculum of synovial fluid tested negative for bacteria and fungi; however, a number of crystals were seen. Based on the result of smear speculum, we suspected the pathology as crystal deposition disease. Polarized light microscopy revealed monocle or triclinic intracellular crystal with a positive birefringence (Figure 5(b)). The patient was diagnosed as having a pseudogout attack of the lumbar spine.

**Figure 6:**
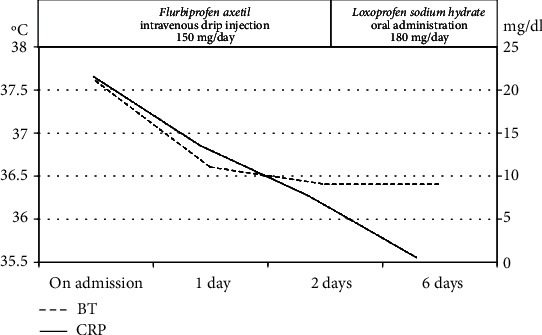
Flurbiprofen axetil was administered by intravenous drip injection for 3 days, and local and systemic inflammatory signs dramatically improved.

Flurbiprofen axetil (nonsteroidal anti-inflammatory drugs) was started immediately by intravenous drip injection and continued for 3 days. Local and systemic indications of inflammation, as well as neurological deficit, dramatically improved within 3 days. Loxoprofen sodium hydrate was administered orally for 8 weeks (Figure 6).

A follow-up evaluation 8 and 12 months after treatment showed complete resolution of symptoms. MR images revealed no abnormal lesion.

## 3. Discussion

Most reported cases of spinal CPPD involve nodular deposition in the ligamentum flavum or atlanto-occipital ligament rather than in the fibrocartilage of a disc [13]. In the lumbar spine region, chronic radiculopathy or cauda equina syndrome by nodular CPPD crystal deposition in the lumbar ligamentum flavum or facet joint has been observed. Most patients are usually treated surgically for conventional lumbar spinal stenosis.

In a search of clinical articles, based on PubMed, since the year 2000, only a few case reports [5, 7, 8, 10, 12, 13] of CPPD crystal deposition involving the lumbar spine were found. Specifically, only 2 case reports described acute CPPD crystal arthritis of the lumbar spine (Table 1) [8, 13].

In our case, there was a large extent of CPPD crystal deposition in the L2-3-4 ligamentum flavum, the L4 spondylolysis, the L3-4 facet joints, and the L3-4 interspinous space. Moreover, calcification in major peripheral joints, right elbow, bilateral hips, left knee, and left ankle, was seen in plain radiographs retrospectively. Although the association with an acute pseudogout attack in the lumbar spine is not confirmed, the patient had a respiratory infection before the onset of neurological symptoms. Generally, CPPD patients are asymptomatic before an acute episode. Acute attacks are typically triggered by intercurrent internal events [15–17]. We hypothesized that the respiratory infection might be one of the initiating factors that activated preexisting asymptomatic CPPD. CPPD crystal arthritis can present as an acute monoarthritis that affects a large joint and produces systematic symptoms such as fever, chills, and malaise. Therefore, septic arthritis must be always ruled out.

The therapeutic modalities for an acute attack of CPPD crystal arthritis were controversial [18]. In our case, multiple cultures of the spinal effusion led to a definitive diagnosis, and 8 weeks of NSAID administration provided the satisfactory recovery from the symptoms.

## 4. Conclusions

We encountered the rare case of an acute attack of pseudogout with the wide lesion in the lumbar spondylolytic spondylolisthesis. Multiple culture of the effusion provided a definitive diagnosis, which allowed for appropriate, minimally invasive treatment for 8 weeks of NSAID administration that provided the satisfactory recovery from the symptoms.

## Figures and Tables

**Table 1:** The articles that describe about acute CPPD crystal arthritis of the lumbar spine.

Studies	Sex/age	Duration of symptoms	Precedent infection	Fever	Neurologic deficit	Level of involvement	Joint CPPD deposition	Treatment
Lee et al. [8]	M/59	2 days	Unknown	No	Yes	L4-5 disc	Unknown	Surgery
Fujishiro et al. [13]	F/71	2 days	Unknown	Yes	No	L4-5 facet	Yes	Aspiration
Our case	F/86	1 day	Yes	Yes	Yes	L3-4-5 LF, L4 lysis, L3-4 interspinous	Yes	NSAIDs

Lysis: spondylolysis.
